# Development of Clustered Resistance Gene Analogs-Based Markers of Resistance to* Phytophthora capsici* in Chili Pepper

**DOI:** 10.1155/2019/1093186

**Published:** 2019-01-03

**Authors:** Nayoung Kim, Won-Hee Kang, Jundae Lee, Seon-In Yeom

**Affiliations:** ^1^Department of Agricultural Plant Science, Division of Applied Life Science (BK21 Plus Program), Gyeongsang National University, Jinju 52828, Republic of Korea; ^2^Institute of Agriculture & Life Science, Gyeongsang National University, Jinju 52828, Republic of Korea; ^3^Department of Horticulture, Chonbuk National University, Jeonju 54896, Republic of Korea

## Abstract

The soil-borne pathogen* Phytophthora capsici* causes severe destruction of* Capsicum* spp. Resistance in* Capsicum* against* P. capsici *is controlled by numerous minor quantitative trait loci (QTLs) and a consistent major QTL on chromosome 5. Molecular markers on* Capsicum* chromosome 5 have been developed to identify the predominant genetic contributor to resistance but have achieved little success. In this study, previously reported molecular markers were used to reanalyze the major QTL region on chromosome 5 (6.2 Mbp to 139.2 Mbp). Candidate resistance gene analogs (RGAs) were identified in the extended major QTL region including 14 nucleotide binding site leucine-rich repeats, 3 receptor-like kinases, and 1 receptor-like protein. Sequence comparison of the candidate RGAs was performed between two* Capsicum* germplasms that are resistant and susceptible, respectively, to* P. capsici. *11 novel RGA-based markers were developed through high-resolution melting analysis which were closely linked to the major QTL for* P. capsici* resistance. Among the markers, CaNB-5480 showed the highest cosegregation rate at 86.9% and can be applied to genotyping of the germplasms that were not amenable by previous markers. With combination of three markers such as CaNB-5480, CaRP-5130 and CaNB-5330 increased genotyping accuracy for 61* Capsicum* accessions. These could be useful to facilitate high-throughput germplasm screening and further characterize resistance genes against* P. capsici *in pepper.

## 1. Introduction

Hot pepper (*Capsicum* spp.) is an economically important crop that belongs to the* Solanaceae* family along with tobacco, potato, and tomato. Hot pepper provides many essential vitamins, and capsaicin is used as a major spicy flavoring in most global cuisines [1]. In 2016, the main pepper-producing countries grew 38 million tons on about 3.7 Mha [2]. The world production and trade value of hot pepper consistently increased during the last decade.

Pathogenic fungi, oomycetes, bacteria, and viruses cause economic damage and crop loss. Pepper production in negatively impacted by approximately 87 different pathogens and diseases [3]. Among them,* Phytophthora capsici* is a highly destructive, broad-host-range oomycetes that was first described in pepper in New Mexico in 1922 [4]. The host range of* P. capsici* includes Solanaceae, Cucurbitaceae, lima beans, and other plants.* P. capsici* is found in North America, South America, Asia, Africa, and Europe.* P. capsici* causes severe disease symptoms such as foliar blight, stem blight, and root, stem, fruit, and foliar rot. The economic impact of* P. capsici* on worldwide vegetable production has been valued at over one billion dollars per year [5].

*P. capsici* forms oospores that survive for years in the soil and can migrate through water and wind during warm (25–28°C) and wet conditions such as those during rainy seasons [6]. After* P. capsici* becomes established at a location, it can be very difficult to control. There is no effective chemical and agricultural strategy. Resistance against* P. capsici* has been reported in cultivated peppers including cultivars ‘AC2258,' ‘PI201232,' ‘PI201234,' and ‘CM334' [7, 8]. Among the resistant cultivars,* Capsicum annuum* ‘CM334' shows a very high degree of resistance to multiple races of* P. capsici *and reported resistance to root rot, crown rot, fruit rot, and foliar blight [9–11].

Previous studies indicated that resistance of pepper to* P. capsici* is under polygenic control governed by complex quantitative trait loci (QTLs) [12–15]. Identification and QTL mapping of genes conferring partial resistance against* P. capsici* in pepper is essential for breeding cultivars with* P. capsici* resistance. The majority of QTLs for* P. capsici* resistance are conserved on* Capsicum *chromosome 5. Numerous molecular markers have been developed within the major QTL region, but little progress has been made in developing functional gene-based markers of* P. capsici *resistance, and no* P. capsici*-resistance gene (*R *gene) has yet been characterized. Recently, high-quality* de novo* sequenced* Capsicum* genomes, including the ‘CM334' genome, were reported [16, 17]. This genomic information from the hot pepper could be applied to plant breeding and the selection of crops with specific traits such as disease resistance or large fruit size [16, 18, 19]. The development of functional gene-based markers and the further characterization of genes that confer resistance against* P. capsici* is key factor for efforts to breed disease-resistant pepper cultivars.

Plants have multiple layers of defense against pathogen attacks, including preformed barriers for continuous defense and programmed immune responses based on pathogen recognition [20–22]. Resistance gene analogs (RGAs) are proteins involved in plant immune responses. RGAs have highly conserved structures, which include nucleotide binding site leucine-rich repeat (NBS-LRR) proteins, receptor-like proteins (RLPs), and receptor-like kinases (RLKs) [21, 23, 24]. RGAs have been cloned as resistance genes in various plants such as wheat (*Lr10 to pathogen*), barley (*Mla6*), rice (*Xa1*), maize (*Rp1-D*), Arabidopsis (*RPM1*), lettuce (*Rgc2*), tobacco (*N*), and tomato (*Prf*) [25–32]. RGAs can be used to identify and characterize *R* genes.

In this study, the sequences of previously developed markers were screened to extend the major QTL region on* Capsicum* chromosome 5 and the RGAs were reanalyzed within that region to identify candidate resistance genes. Marker development on candidate genes was performed by comparing single nucleotide polymorphisms (SNPs) between the* P. capsici*-resistant cultivar* C. annuum* ‘CM334' and the* P. capsici*-susceptible cultivar* C. annuum* ‘Daepoongcho'. A total of 61* Capsicum* accessions were used to validate the newly developed RGA-based markers through high-resolution melting (HRM) analysis. These markers could be useful to validate the genotyping of germplasms and to further characterize resistance genes against* P. capsici*.

## 2. Materials and Methods

### 2.1. Plant Materials

61* Capsicum* accessions (44 resistant, 3 moderately resistant, and 14 susceptible cultivars; Table S1) [33–38] were used to validate cosegregation of molecular markers. All plants were grown in 32-cell trays filled with soil. The plants were kept in a growth chamber at 25°C with a 16 h/8 h (light/darkness) photoperiod.

### 2.2. Pathogen Preparation and Plant Inoculation with P. capsici

The preparation of the* P. capsici* inoculum was described in previous study [11].* P. capsici* was grown for 7 days in potato dextrose agar medium at 27°C and mycelial plugs (6 mm in diameter) were transferred on V8 agar medium for zoospore production. After 5 days, the mycelia grown on V8 agar medium were damaged using spreader and incubated under fluorescent lights for 2 days. The zoosporangia were shocked by incubating at 4°C with sterile water for 1 h 30 min to initiate release zoospores, followed by 30 min at 28°C for equilibration. The zoospores were collected and the concentration was adjusted to 1 × 10^5^ zoospores · mL^−1^ counted by hemocytometer, and 2 mL of suspension was drenched at the root of each six-true-leaf stage of pepper plant. The inoculated pepper plants were kept at 25°C with a 16-h light photoperiod condition. Evaluation of disease symptoms was assessed using disease index of 0 to 3 scale (0 = no symptom; 1 = leaf wilting but no necrosis or less than 30% of the leaf wilted; 2 = leaf wilting and slightly necrosis stem or less than 60% of the leaf wilted; 3 = plant dead) [37]. The classification of the pepper lines, as resistant, moderately resistant or susceptible, was made based on the average disease index of each line, where if the scale was < 1, the pepper line was considered as resistant (R), 1 ≤ disease index < 2 confer as moderately resistant (MR), and if the scale was < 3 as susceptible (S). The results of the phenotype correspond to those as shown in Kim* et al*., 2017 [38].

### 2.3. Genomic DNA Extraction

Genomic DNA was extracted from the young leaves of plant samples using a slightly modified cetyltrimethylammonium bromide (CTAB) method [39]. First, the leaves were grinded using a pestle and mixed them with CTAB buffer, polyvinylpyrrolidone, and *β*-mercaptoethanol. The samples were incubated at 65°C for 1 h, added chloroform with isoamyl alcohol (24:1), and centrifuged them at 4°C 15,814* g* for 15 min. Supernatant was transferred to a new 1.5 mL tube and incubated it for 30 min at −20°C with isopropyl alcohol. The precipitated genomic DNA was washed using 70% ethanol and centrifuged it at 4°C 15,814*g* for 10 min. DNA pellet was dissolved in deionized water and treated it with ribonuclease A. Concentration of genomic DNA was measured by a NanoDrop™ Spectrophotometer (ND-2000, ThermoFisher Scientific, Waltham, MA, USA) and then diluted the sample to a final DNA concentration of 20 ng·*μ*L^−1^.

### 2.4. Major QTL-Related Marker Analysis

The DNA sequences of 5 simple sequence repeat (SSR), 8 cleaved amplified polymorphic sequence (CAPS), and 18 SNP markers from the published maps of pepper chromosome 5 were used and compared with the* C. annuum* ‘CM334' genome version 1.55 (http://genome.pepper.snu.ac.kr/) using BLASTn. The major QTL region on chromosome 5 was extended including all of the previously identified molecular markers and then candidate resistance genes from the extended major QTL region were selected.

### 2.5. Candidate Gene Selection and High-Resolution Melting Analysis

RGAs were identified according to the domain structure of the genes in the extended major QTL region. Domain structure analysis was conducted using SMART (http://smart.embl-heidelberg.de/) and Pfam (http://pfam.xfam.org/). The RGA sequences were expanded including an additional 1 kb from both ends of the sequences using an in-house pipeline. A multiple sequence alignment tool MUSCLE (http://www.ebi.ac.uk/) was used to identify SNPs between* C. annuum* ‘CM334' and* C. annuum* ‘Daepoongcho' for developing RGA-based markers. The candidate genes were selected based on the locations of the SNPs within the expanded QTL region.

Peptide and DNA sequences were downloaded from the Pepper Genome Platform (http://genome.pepper.snu.ac.kr/) and designed primers using primer3plus (http://www.bioinformatics.nl/cgi-bin/primer3plus/primer3plus.cgi; Table 1) for HRM analysis of molecular markers. HRM analysis was performed to validate the cosegregation of the newly developed RGA-based markers among 61* Capsicum* accessions using LightCycler® Real-Time PCR (Roche, Basel, Switzerland). The reaction solution had a total volume of 20 *μ*L and contained 40 ng genomic DNA, 1 *μ*L each of two primers at 10 pmol·*μ*L^−1^, 0.1 *μ*L EasyTaq® DNA polymerase (Transgene Biotech, Beijing, China), 2 *μ*L 10× EasyTaq® buffer (Transgene Biotech, Beijing, China), 1 *μ*L 2.5 mM dNTPs (Transgene Biotech, Beijing, China), 1 *μ*L SYTO®9 green fluorescent nucleic acid stain (Life Technologies, Carlsbad, CA, USA), and sterilized water. The PCR conditions were 5 min denaturation at 95°C followed by 10 s at 95°C and annealing with extension 20 s at 60°C for 40 cycles. The melting curve stage was a progression of 95°C for 1 min, 40°C for 1 min, and 65°C for 1 s with fluorescence estimated at 0.2°C intervals. Genotyping of the molecular markers was analyzed through High-Resolution Melt software version 1.1 (Roche, Basal, Switzerland). 

## 3. Results and Discussion

### 3.1. Candidate RGA Markers with Integration of Genetic and Genomic Data on Pepper Chromosome 5

Genetic and genomic data on pepper chromosome 5 were integrated to determine the extended region of a major resistance QTL. The following DNA sequences and QTL information were used in this study: CAPS markers mapped to the* Pc.5.2, Pc.5.3 *region of ‘H3' ×  ‘Vania' (HV), and ‘Perenial' ×  ‘Yolo Wonder' (PY) [40]; SNP markers within the* Pc5.1* region of* ‘*Early Jalapeno*'* ×  ‘CM334' (EC), the* Phyto5* region of ‘YCM334' ×  ‘Tean,' and the* Pc5.2* region of ‘CM334' ×  ‘Chilsungcho' (CC) and ‘NB1' ×  ‘Bhut Jolokia' [41–44]; and SSR markers mapped to the* Pc5.1 *region of ‘Manganji' ×  ‘CM334' [45]. The genomic locations of the previously developed markers were identified using BLASTn with the* C. annuum* ‘CM334' genome. The primer sequences and the genomic location of several representative markers are shown in Table 1.

**Figure 1:**
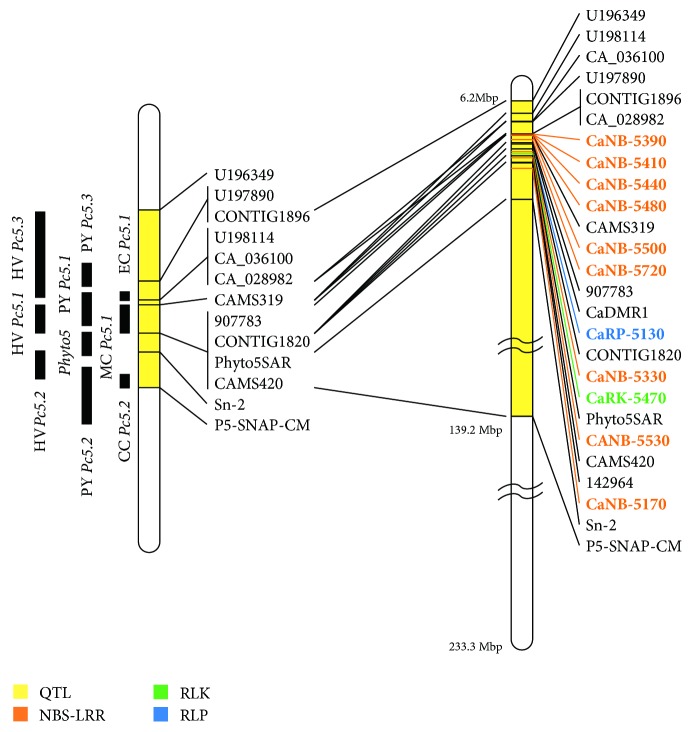
Integration of genetic and genomic data on chromosome 5. The genetic map of chromosome 5 is on the left, and the physical map is on the right. Quantitative trait loci (QTL) of the previously developed markers are shown on left side of the genetic map. The physical map is drawn to show the actual chromosome size, which is 233.3 Mbp in ‘CM334.' The extended QTL region spans from 6.2 Mbp to 139.2 Mbp and in shown in yellow color. The other colors indicate information about the RGA-based markers within the QTL region, which are shown on the right side of the physical map. Previously developed markers are integrated with the genetic map and physical map with their actual locations on chromosome 5. Orange: NBS-LRR-based marker, green: RLK-based marker, and blue: RLP-based marker.

A physical map of chromosome 5 was drawn including the locations of the markers and obtained the extended major QTL region for the selection of candidate resistance genes. The extended QTL region spanned from 6.2 Mbp (U196349) to 139.2 Mbp (P5-SNAP-CM) and contained 10 QTLs and 845 genes from 56 scaffolds (Table 1 and Figure 1). The genes included NB-ARC domain containing protein, GRAS family transcription factor, cytochrome P450, and proteins of unknown function.

Using the domain analysis tool, 18 RGAs in the extended QTL region including 14 NBS-LRR proteins, 3 RLKs, and 1 RLP were identified (Table S2). Previously, Rehrig* et al*., 2014, reported several RGAs nearby QTL peak on chromosome 5 using ‘CM334' genome (ver.1.5), but they did not developed RGA-based markers to evaluate* P. capsici*-resistance resources. In this study, additional five RGAs were identified from the updated ‘CM334' genome (ver. 1.55) as shown in Table S2. The 14 NBS-LRR proteins consisted of seven partial type NBS-LRRs and seven full type NBS-LRRs. All three RLKs had a kinase-TM-kinase domain. The RLP had a signal peptide-LRR domain. The majority of candidate genes that have been cloned as *R* genes in plant species are RGAs. To date, more than 314 functional *R* genes have been identified in plants [46]. Among them, 80% encode NBS-LRR proteins (191/314) or RLPs/RLKs (60/314). In* Solanaceae*, all of the cloned *R* genes against* Phytophthora infestans *were identified RGAs such as* Rpi-blb1*,* Rpi-blb2*,* R2*,* R3a,* and* ELR* [47–51]. Therefore, the identification of RGAs and development of RGA-based markers could be useful in the further characterization of* P. capsici*-resistance genes.

### 3.2. Development of SNP Markers

**Figure 2:**
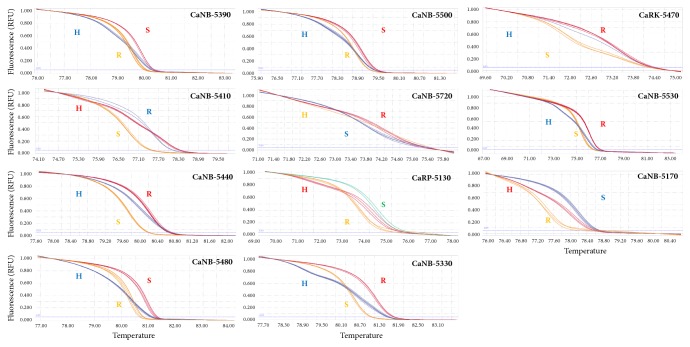
High-resolution melting (HRM) curves of the newly developed markers. Sixty-one* Capsicum* accessions were used to perform HRM analysis, and curves represent several germplasms as genotypes. The x-axis indicates temperature, and the y-axis shows fluorescence. R: resistant homozygote, H: resistant heterozygote, and S: susceptible homozygote.

Eighteen RGAs were reanalyzed to identify SNPs between resistant and susceptible* P. capsici* germplasms by multiple sequence alignment. Among the 18 RGAs, 11 had SNPs and were selected as candidate genes for the development of molecular markers (Table 2 and Table S2). The RGA-based markers had four SNP types (A/C, A/G, T/C, and T/G) and had from one to three SNPs between the resistant and susceptible germplasms (Table 2). Using HRM analysis, nine NBS-LRR-based markers (CaNB-5390, CaNB-5410, CaNB-5440, CaNB-5480, CaNB-5550, CaNB-5720, CaNB-5330, CaNB-5530, and CaNB-5170), one RLK-based marker (CaRK-5470), and 1 RLP-based marker (CaRP-5130) were developed. The HRM curves clearly distinguished among three genotypes for each of the markers: resistant homozygous, resistant heterozygous, and susceptible homozygous (Figure 2).

**Figure 3:**
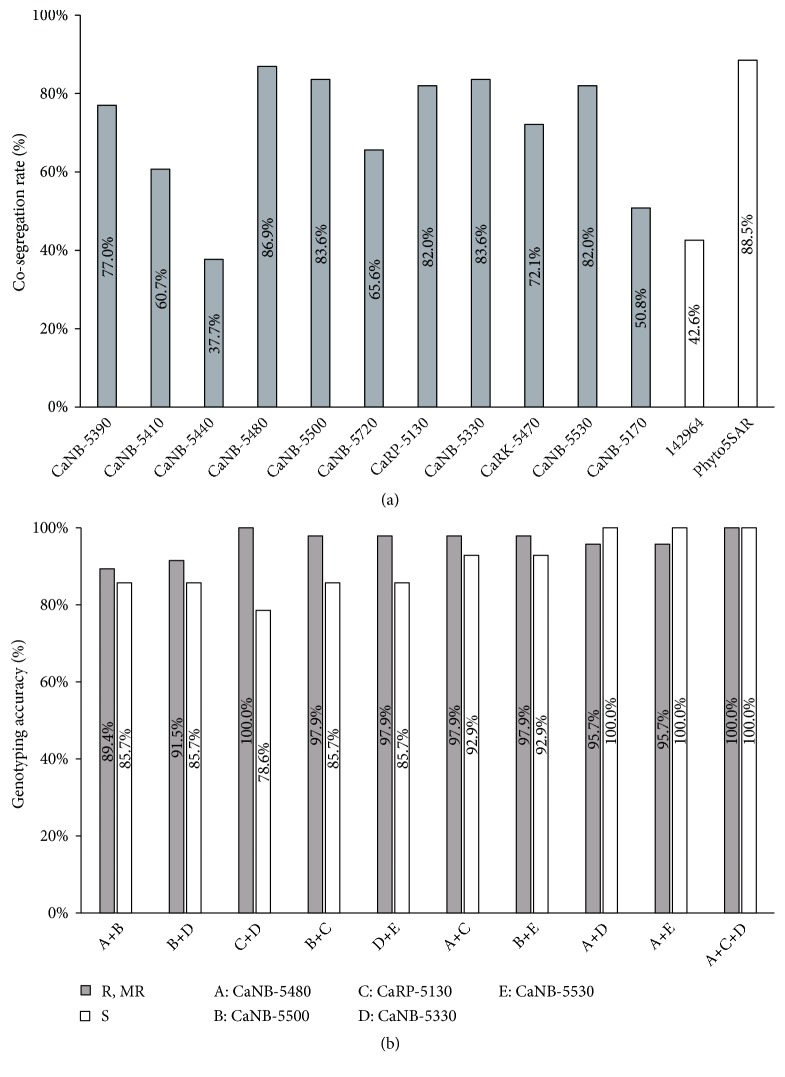
Cosegregation rate obtained by genotyping RGA-based markers in 61 cultivars. (a) The cosegregation rate of the each marker. The x-axis shows the names of the molecular markers. The y-axis shows the cosegregation rate. Newly developed markers are indicated by grey bars. White bars show previously developed markers 142964 and Phyto5SAR. (b) The genotyping accuracy when applied with combination of RGA markers at 61 cultivars. The x-axis shows combination of the markers and y-axis shows genotyping accuracy. Grey bars show genotyping accuracy of resistant and moderately resistant cultivars. Genotyping accuracy of susceptible cultivars is indicated by white bars.

The RGA-based SNP markers were applied to genotyping of 61* Capsicum* accessions with previously developed markers such as 142964 [42] and Phyto5SAR [43]. In a previous report, the region spanning from 20.2 Mbp to 29.29 Mbp on chromosome 5 was reported as the major core QTL region [43]. Phyto5SAR was closely linked to the major core QTL, and was used to assess the genotyping accuracy of the new developed markers. The genotyping results of the newly developed markers are shown on Table 3 and Table S3, which were divided into four groups as genotyping accuracy on cultivars. The phenotypes in cultivars belong to Group 1 and Group 2 were matched with genotyping results by six markers (Table 3). The cosegregation rates varied from 37.7% to 86.9% (Figure 3(a)). Several markers adjacent to Phyto5SAR had a tendency to show a high cosegregation rate, which were CaRP-5130 (82%), CaNB-5330 (83.6%), CaRK-5470 (72.1%), and CaNB-5530 (82%). Although far from Phyto5SAR on the physical map, CaNB-5480 showed the highest cosegregation rate (86.9%) except for eight cultivars (13.1%). CaNB-5480 could be applicable to genotyping of germplasms that are not amenable to genotyping (R31-33; R35-37) using Phyto5SAR. In a previous study, the combination of molecular markers increased genotyping accuracy of* Cf-9 *locus in tomato cultivars [52]. In our study, by combining two or three markers, the genotyping accuracy was increased compared to that in single RGA maker (Figure 3). Combination of three markers such as CaNB-5480, CaRP-5130, and CaNB-5330 cosegregated with the resistance and susceptible phenotypes in pepper accessions used in this study (Figure 3(b)). Genotyping results of CaNB-5480 and Phyto5SAR showed 98.4% genotyping accuracy except for one cultivar (R43) and CaNB-5130 can apply to the genotyping of the R43 cultivar. The genotyping accuracy of combination of two or three markers ranged from 78.6% to 100% (Figure 3(b)). Taken together, combination with highly linked markers could be used efficiently for genotyping of pepper varieties for further pepper breeding.

The variation of the cosegregation rate (37.7% to 86.9%) suggested the conversion of the major QTL region between the genetic and physical maps. Kim and associates [17] constructed pseudomolecules using a high-density map with 6,281 markers derived from* C. annuum *‘Perennial' and* C. annuum *‘Dempsey'. Scaffold anchoring was conducted with genetic maps of a cross between* C. annuum* ‘NuMexRNAKY' and* C. frutescens* acc. 2814-6 [53]. Neither previous study used ‘CM334'-related populations. Genomic variation between ‘CM334' and other pepper germplasms could represent differences in cosegregation [54]. Further reanalyses of the pepper genomic structure could reveal reanchoring or rearrangement within or among chromosomes.

To date, several markers linked to the major resistance QTL in pepper have been developed including restriction fragment length polymorphisms (RFLPs), randomly amplified polymorphic DNA (RAPD), amplified fragment length polymorphisms (AFLPs), and SNPs, which could lead to marker-assisted selection for breeding* P. capsici*-resistant lines [55, 56]. Wang and associates [57] also developed SSR markers that tightly linked to the resistance gene in pepper line ‘PI201234'. However, the previously developed markers from the different genetic maps could be insufficient to determine whether the QTL region includes a QTL that is conserved among different progenies.* P. capsici* isolates with different virulence factors and/or inoculation concentrations also have variable disease phenotypes, which cause variation in cosegregation rates of molecular markers [43, 57]. Rehrig and associates [44] reported cosegregated* CaDMR1* with QTL* Pc5.1* as a candidate for resistance to* P. capsici* in pepper, but its function was not determined yet. Here, newly closely linked RGA-based markers of* P. capsici* resistance were developed, which can be used to genotype breeding sources and to further characterize *R* genes. Our markers may be used in combination with other markers such as CaNB-5480, CaRP-5130, and CaNB-5330 to efficiently determine the phenotypes of pepper germplasms. Such data would also be sufficient to determine the resistance gene spectrum in the QTL region on chromosome 5.

## 4. Conclusion

In this study, 11 novel RGA-based markers were developed that are linked to major QTL for* P. capsici* resistance. Among the markers, CaNB-5480 showed the most closely linked marker to major QTL. With combination of CaNB-5480, CaRP-5130 and CaNB-5330 provide the most accurate assessment of genotyping among 61* Capsicum* accessions. Together, as combination with other markers, it could be more efficiently phenotyping of pepper germplasms and to further characterize resistance genes against* P. capsici.*

## Figures and Tables

**Table 1:** Information about the molecular markers within the extended major QTL region on chromosome 5.

Marker	QTL	Genome location (Mbp)	Primer sequences		References
			Forward primer (5′-3′)	Reverse primer (5′-3′)	
U196349	HV *Pc5.3*	6.17	CCTGGGAGAGGAGTCCTACA	GCAAGAAACAGCGCCTTTAG	[40]
U198114	HV *Pc5.3*	11.35	TTGGGCTCAATTAAACCATACA	GCACCCCTTGATTGAGAGAA	[40]
CA_036100^†^	EC *Pc5.1*	14.79	GTATATATTTATCAAAATTAATAATCTAGAGAGTATTGAGAGTG­CTTAGTGAGAGAGATTAGCAAATTAGCAACAATGGGTAAATTGATTTTTGTAGCAAT		[44]
U197890	PY *Pc5.3*	14.93	CCATATGGTCCTCCTCCAGA	CTTTCAACCACTTCGGCAAT	[40]
CONTIG1896	*Phyto5*	19.99	CATAACACAACCCAATTGCAGAACC	GTCCTACACTTGTTACAGCTGCC	[43]
CA_028982^†^	EC *Pc5.1*	20.02	GTGGAGGACGAATGTGGTATTTCTCAACCCCAACAAAGAGAAAAAAAACACGTAAAAATAATTTTGAGGAACTAAGGATAAGAAATGCAACAATGGAAGGC		[44]
CaNB-5390		20.2	AAGCCGGGTCTTATAAACTTCCAT	CTTGTGAACATCCAAAGTTAGGGG	In this study
CaNB-5410		20.25	TAGAATCCATCAACCGTACGTAGA	ATTGAGTTAGGTGGCATCTTGACT	In this study
CaNB-5440		20.3	GTTCGACGAGGCTCTACTTTGA	TCAGTCTGCCAGGATTAACAATGA	In this study
CaNB-5480		20.51	TCGAAATCAATACTCCTCCTTCCC	CTCACGCGTTGTTCTTAAAAGGTT	In this study
CAMS319	MC *Pc5.1*	20.57	CATAACACAACCCAATTGCAGAACC	CATAACACAACCCAATTGCAGAACC	[45]
CaNB-5500		20.79	TCATTTATGCGTGCAGTATCGTAC	AAATATACCCCTCAATGACACACA	In this study
CaNB-5720		22.5	GGAAGTCCATTTTATCTATCCTCAGA	ATGATTTTGGGGGATTTCAATAGT	In this study
907783	*Phyto5*	23.78	GACTCCATTTTCATGTTAGCGTGC	CTTGCACCTGGTAAACAACGC	[43]
CaRP-5130		26.43	GACACATTTTGCAGATTCATCAAC	CACCCAAAAAGGTAAAAAGAAACA	In this study
CONTIG1820	*Phyto5*	26.44	GCAAGGAGAAATCAACAGAGCC	AATACAAAGAACGCAGTAGGAGGG	[43]
CaNB-5330		27.63	AAGAAAGCCGTCACCTTCATAGAT	GCTAATTTGCAAGGATTACGCTCA	In this study
CaRK-5470		28.5	TTTCAATATCCAAAGAACACAACA	GTGAACCCAATGTGATAGTGAAAG	In this study
Phyto5SAR	*Phyto5*	29.29	GGCACAACAATAGTCACAACGG	GAGACTAAGAAAGTTGGACGCC	[43]
CaNB-5530		30.19	GCTCCGTGTTCATCTATGTTAAACA	AACAGAAGGATCCATCAGCATCAT	In this study
CAMS420	MC *Pc5.1*	32.04	CATAACACAACCCAATTGCAGAACC	CATAACACAACCCAATTGCAGAACC	[45]
142964		32.29	AAAGCTTTTTACATCTCATTCAACG	AGTTTGAGCAACATAGATGTGGAG	[42]
CaNB-5170		34.56	TATCTCCTACATCCCACTGACAAC	CTTGTCATGGGTGGTGCATTAATG	In this study
Sn-2	HV *Pc5.2*	47.72	TTCGATCCACACCATCATCT	TCCTTCAATGGCTTTCCATC	[40]
P5-SNAP-CM	CC *Pc5.2*	139.2	TCATGAGGTTGCTATTAAGATTGGTCCTGTTATATA	CATAGAAAGGGATATCATCTGGTACATGCAGAAA	[41]

Table 1 contains information about newly developed markers and several representative markers among the 31 previously developed markers. ^†^Primers for CA_036100 and CA_028982 are flanking sequences of CM334.

**Table 2:** Comparison of SNPs between *P. capsici*-resistant and *P. capsici*-susceptible germplasms.

Marker	‘CM334' allele^‡^	‘Daepoongcho' allele^§^	Flanking sequence
CaNB-5390	T	C	AAGCCGGGTCTTATAAACTTCCATAGTGGTTTCCAACATGG­CATAATACATAAAAATGCCCTT(T/C) ^¶^AACTCGTCCTCAA­ATCACACCTACGACCCCTAACTTTGGATGTTCACAAG

CaNB-5410	G, G	T, A	TAGAATCCATCAACCGTACGTAGAT(G/T)CTAAGTTTTT(G/A)T­TTTTTTATGAAGAAGAAAAAAGTCACAGTTCTAGTCAAG­ATGCCACCTAACTCAAT

CaNB-5440	G, C	A, T	GTTCGACGAGGCTCTACTTTGAGAAGTTCAGCTA(G/A)CAATC­TTTCTACAA(C/T)ATCCAGTAAGCTGGGAGGTAGTCCTCCT­CATTGTTAATCCTGGCAGACTGA

CaNB-5480	T, T, G	C, C, A	TCGAAATCAATACTCCTCCTTCCCTAGGGGGTCAGTTGACAGGTAA­TTAAATTCCCAACCTCCAA(T/C)ACATTTTTCAGT­CAAAGGGTAAAGGGTGGGACTTTTTTTAC(T/C)GCGT­AAGGTAAATTAGTCTCCCCGCCTTAC(G/A)CTTTATCATTA­GCTAATGTCATTAACCTTTTAAGAACAACGCGTGAG

CaNB-5500	T	G	TCATTTATGCGTGCAGTATCGTACTCTCATGCAAGACTTATATC­AGTGAAC(T/G)TATGCATTGAAGCATAACTTGTACCACTTAT­AAACTTATGTCCTGGAGCAGAATGTGTGTCATTGAGGGGTATATTT

CaNB-5720	A	G	GGAAGTCCATTTTATCTATCCTCAGAA(A/G)TGAGTACACTATTGAA­ATCCCCCAAAATCAT

CaRP-5130	A	G	GACACATTTTGCAGATTCATCAACTAA(A/G)TTGCTGTTTCTTTTT­ACCTTTTTGGGTG

CaNB-5330	C, G	T, A	AAGAAAGCCGTCACCTTCATAGATGATGGGTTGTCGATGGG­C(C/T)CCCTCACTACATAAGTCCACATTGGTAC(G/A)TTAAT­GGGGATTTGAGCGTAATCCTTGCAAATTAGC

CaRK-5470	G	A	TTTCAATATCCAAAGAACACAACA(G/A)TTAATATTCTTTTCTTTTCTTT­CACTATCACATTGGGTTCA

CaNB-5530	C	T	GCTCCGTGTTCATCTATGTTAAACAAGATTTCTTGCAA(C/T)ATGTCATGA­TGCTGATGGATCCTTCTGTT

CaNB-5170	A	C	TATCTCCTACATCCCACTGACAACAGTTAGCTTTATATTGTCAACTTTA­TCTATC(A/C)AGACTCTGTTTTTCAGTCACTTTATT­GTCCATTAATGCACCACCCATGACAAG

^‡^ *P. capsici*-resistant germplasm *C. annuum* ‘CM334'; ^§^*P. capsici*-susceptible germplasm *C. annuum *‘Daepoongcho';  ^¶^(CM334 allele/Daepoongcho allele).

**Table 3:** The genotypes of the major QTL for *P. capsici* resistance linked SNP markers.

Group	Code^†^	Marker					
		CaNB-5480	CaNB-5500	CaRP-5130	CaNB-5330	Phyto5SAR	CaNB-5530
Group 1	R1	R ^*∗*^	R ^*∗*^	R ^*∗*^	R ^*∗*^	R ^*∗*^	R ^*∗*^
	R2	H ^*∗*^	H ^*∗*^	H ^*∗*^	H ^*∗*^	H ^*∗*^	H ^*∗*^
	R3	R ^*∗*^	R ^*∗*^	R ^*∗*^	H ^*∗*^	H ^*∗*^	R ^*∗*^
	R4	H ^*∗*^	H ^*∗*^	H ^*∗*^	H ^*∗*^	H ^*∗*^	H ^*∗*^
	R5	H ^*∗*^	H ^*∗*^	H ^*∗*^	H ^*∗*^	H ^*∗*^	H ^*∗*^
	R6	H ^*∗*^	H ^*∗*^	H ^*∗*^	H ^*∗*^	H ^*∗*^	H ^*∗*^
	R7	H ^*∗*^	H ^*∗*^	H ^*∗*^	H ^*∗*^	H ^*∗*^	H ^*∗*^
	R8	H ^*∗*^	H ^*∗*^	H ^*∗*^	H ^*∗*^	H ^*∗*^	H ^*∗*^
	R9	H ^*∗*^	H ^*∗*^	H ^*∗*^	H ^*∗*^	H ^*∗*^	H ^*∗*^
	R10	H ^*∗*^	H ^*∗*^	H ^*∗*^	H ^*∗*^	H ^*∗*^	H ^*∗*^
	R11	R ^*∗*^	R ^*∗*^	H ^*∗*^	H ^*∗*^	H ^*∗*^	H ^*∗*^
	R12	R ^*∗*^	R ^*∗*^	H ^*∗*^	H ^*∗*^	H ^*∗*^	H ^*∗*^
	R13	R ^*∗*^	R ^*∗*^	H ^*∗*^	H ^*∗*^	H ^*∗*^	H ^*∗*^
	R14	R ^*∗*^	R ^*∗*^	R ^*∗*^	H ^*∗*^	H ^*∗*^	H ^*∗*^
	R15	R ^*∗*^	R ^*∗*^	R ^*∗*^	H ^*∗*^	H ^*∗*^	H ^*∗*^
	R16	R ^*∗*^	R ^*∗*^	R ^*∗*^	H ^*∗*^	H ^*∗*^	R ^*∗*^
	R17	R ^*∗*^	R ^*∗*^	R ^*∗*^	H ^*∗*^	R ^*∗*^	H ^*∗*^
	R18	R ^*∗*^	R ^*∗*^	H ^*∗*^	H ^*∗*^	H ^*∗*^	H ^*∗*^
	R19	R ^*∗*^	R ^*∗*^	R ^*∗*^	H ^*∗*^	H ^*∗*^	H ^*∗*^
	R20	R ^*∗*^	R ^*∗*^	R ^*∗*^	R ^*∗*^	R ^*∗*^	R ^*∗*^
	R21	H ^*∗*^	H ^*∗*^	H ^*∗*^	H ^*∗*^	H ^*∗*^	H ^*∗*^
	R22	H ^*∗*^	H ^*∗*^	H ^*∗*^	H ^*∗*^	H ^*∗*^	H ^*∗*^
	R23	R ^*∗*^	R ^*∗*^	R ^*∗*^	H ^*∗*^	H ^*∗*^	H ^*∗*^
	R24	R ^*∗*^	R ^*∗*^	R ^*∗*^	H ^*∗*^	R ^*∗*^	R ^*∗*^
	R25	H ^*∗*^	H ^*∗*^	H ^*∗*^	H ^*∗*^	H ^*∗*^	H ^*∗*^
	R26	R ^*∗*^	R ^*∗*^	R ^*∗*^	R ^*∗*^	R ^*∗*^	R ^*∗*^
	R27	R ^*∗*^	R ^*∗*^	H ^*∗*^	H ^*∗*^	H ^*∗*^	H ^*∗*^
	R28	H ^*∗*^	H ^*∗*^	H ^*∗*^	H ^*∗*^	H ^*∗*^	H ^*∗*^
	R29	H ^*∗*^	H ^*∗*^	H ^*∗*^	H ^*∗*^	H ^*∗*^	H ^*∗*^
	R30	R ^*∗*^	R ^*∗*^	H ^*∗*^	H ^*∗*^	H ^*∗*^	H ^*∗*^
	MR1	H ^*∗*^	H ^*∗*^	H ^*∗*^	H ^*∗*^	H ^*∗*^	H ^*∗*^
	MR2	R ^*∗*^	R ^*∗*^	H ^*∗*^	H ^*∗*^	H ^*∗*^	H ^*∗*^
	MR3	R ^*∗*^	R ^*∗*^	H ^*∗*^	H ^*∗*^	H ^*∗*^	H ^*∗*^

Group 2	S1	S ^*∗*^	S ^*∗*^	S ^*∗*^	S ^*∗*^	S ^*∗*^	S ^*∗*^
	S2	S ^*∗*^	S ^*∗*^	S ^*∗*^	S ^*∗*^	S ^*∗*^	S ^*∗*^
	S3	S ^*∗*^	S ^*∗*^	S ^*∗*^	S ^*∗*^	S ^*∗*^	S ^*∗*^
	S4	S ^*∗*^	S ^*∗*^	S ^*∗*^	S ^*∗*^	S ^*∗*^	S ^*∗*^

Group 3	R31	H ^*∗*^	H ^*∗*^	H ^*∗*^	H ^*∗*^	- ^*∗∗*^	H ^*∗*^
	R32	H ^*∗*^	H ^*∗*^	H ^*∗*^	R ^*∗*^	S ^*∗∗*^	S ^*∗∗*^
	R33	R ^*∗*^	R ^*∗*^	H ^*∗*^	R ^*∗*^	S ^*∗∗*^	S ^*∗∗*^
	R34	H ^*∗*^	H ^*∗*^	H ^*∗*^	S ^*∗∗*^	H ^*∗*^	H ^*∗*^
	R35	H ^*∗*^	H ^*∗*^	- ^*∗∗*^	R ^*∗*^	S ^*∗∗*^	H ^*∗*^
	R36	H ^*∗*^	H ^*∗*^	S ^*∗∗*^	H ^*∗*^	S ^*∗∗*^	S ^*∗∗*^
	R37	H ^*∗*^	H ^*∗*^	S ^*∗∗*^	R ^*∗*^	S ^*∗∗*^	S ^*∗∗*^
	R38	R ^*∗*^	S ^*∗∗*^	R ^*∗*^	S ^*∗∗*^	R ^*∗*^	R ^*∗*^
	R39	S ^*∗∗*^	R ^*∗*^	H ^*∗*^	H ^*∗*^	H ^*∗*^	S ^*∗∗*^
	R40	S ^*∗∗*^	S ^*∗∗*^	H ^*∗*^	H ^*∗*^	H ^*∗*^	H ^*∗*^
	R41	S ^*∗∗*^	S ^*∗∗*^	R ^*∗*^	S ^*∗∗*^	R ^*∗*^	R ^*∗*^
	R42	S ^*∗∗*^	S ^*∗∗*^	R ^*∗*^	S ^*∗∗*^	H ^*∗*^	R ^*∗*^
	R43	S ^*∗∗*^	S ^*∗∗*^	H ^*∗*^	S ^*∗∗*^	S ^*∗∗*^	S ^*∗∗*^
	R44	S ^*∗∗*^	S ^*∗∗*^	S ^*∗∗*^	R ^*∗*^	R ^*∗*^	R ^*∗*^

Group 4	S5	S ^*∗*^	S ^*∗*^	S ^*∗*^	R ^*∗∗*^	S ^*∗*^	S ^*∗*^
	S6	S ^*∗*^	S ^*∗*^	R ^*∗∗*^	S ^*∗*^	S ^*∗*^	R ^*∗∗*^
	S7	S ^*∗*^	S ^*∗*^	R ^*∗∗*^	S ^*∗*^	S ^*∗*^	R ^*∗∗*^
	S8	S ^*∗*^	S ^*∗*^	R ^*∗∗*^	S ^*∗*^	S ^*∗*^	R ^*∗∗*^
	S9	S ^*∗*^	S ^*∗*^	R ^*∗∗*^	H ^*∗∗*^	S ^*∗*^	S ^*∗*^
	S10	S ^*∗*^	S ^*∗*^	R ^*∗∗*^	H ^*∗∗*^	S ^*∗*^	H ^*∗∗*^
	S11	S ^*∗*^	H ^*∗∗*^	S ^*∗*^	H ^*∗∗*^	S ^*∗*^	S ^*∗*^
	S12	S ^*∗*^	H ^*∗∗*^	R ^*∗∗*^	H ^*∗∗*^	S ^*∗*^	R ^*∗∗*^
	S13	R ^*∗∗*^	R ^*∗∗*^	S ^*∗*^	S ^*∗*^	S ^*∗*^	S ^*∗*^
	S14	H ^*∗∗*^	H ^*∗∗*^	- ^*∗∗*^	S ^*∗*^	S ^*∗*^	S ^*∗*^

Cosegregation genotypes are indicated by *∗*; *∗∗* indicates recombinant genotypes. R: resistant homozygote, H: resistant heterozygote, S: susceptible homozygote, -: not detected. Group 1: genotypes matched with R phenotypes in resistant and moderately resistant cultivars, Group 2: genotypes matched with S phenotypes in susceptible cultivars, Group 3: a group of resistant cultivars with low genotyping accuracy, and Group 4: a group of susceptible cultivars with low genotyping accuracy. ^†^Accession name is shown on Table S1.

## Data Availability

The data used to support the findings of this study are included within the article and supplementary materials.

## Supplementary Materials

Supplementary Materials Supp. Table S1: plant materials used to validate the molecular markers in the study. Supp. Table S2: the information of the reanalyzed RGAs in this study. Supp. Table S3: sequence variation (SNPs) between resistant and susceptible plants in pepper varieties.
